# LINC01559 promotes lung adenocarcinoma metastasis by disrupting the ubiquitination of vimentin

**DOI:** 10.1186/s40364-024-00571-3

**Published:** 2024-02-05

**Authors:** Hao Feng, Dengfei Xu, Chenyang Jiang, Yuming Chen, Junru Wang, Zirui Ren, Xiang Li, Xu Dong Zhang, Shundong Cang

**Affiliations:** 1 Department of Oncology, Henan Provincial International Coalition Laboratory of Oncology Precision Treatment, Henan Provincial Academician Workstation of Non-Coding RNA Translational Research, Henan Provincial People’s Hospital, Zhengzhou University People’s Hospital, Zhengzhou, 450003 Henan China; 2 School of Biomedical Sciences and Pharmacy, The University of Newcastle, Callaghan, NSW 2308 Australia; 3 Translational Research Institute, Henan Provincial and Zhengzhou City Key Laboratory of Non-Coding RNA and Cancer Metabolism, Henan International Join Laboratory of Non-Coding RNA and Metabolism in Cancer, Henan Provincial People’s Hospital, Academy of Medical Sciences, Zhengzhou University, Henan, 450003 China

**Keywords:** Lung adenocarcinoma, lncRNA, LINC01559, Vimentin, Metastasis

## Abstract

**Background:**

Distant metastasis is the major cause of lung adenocarcinoma (LUAD)-associated mortality. However, molecular mechanisms involved in LUAD metastasis remain to be fully understood. While the role of long non-coding RNAs (lncRNAs) in cancer development, progression, and treatment resistance is being increasingly appreciated, the list of dysregulated lncRNAs that contribute to LUAD pathogenesis is also rapidly expanding.

**Methods:**

Bioinformatics analysis was conducted to interrogate publicly available LUAD datasets. In situ hybridization and qRT-PCR assays were used to test lncRNA expression in human LUAD tissues and cell lines, respectively. Wound healing as well as transwell migration and invasion assays were employed to examine LUAD cell migration and invasion in vitro. LUAD metastasis was examined using mouse models in vivo. RNA pulldown and RNA immunoprecipitation were carried out to test RNA–protein associations. Cycloheximide-chase assays were performed to monitor protein turnover rates and Western blotting was employed to test protein expression.

**Results:**

The expression of the lncRNA LINC01559 was commonly upregulated in LUADs, in particular, in those with distant metastasis. High LINC01559 expression was associated with poor outcome of LUAD patients and was potentially an independent prognostic factor. Knockdown of LINC01559 diminished the potential of LUAD cell migration and invasion in vitro and reduced the formation of LUAD metastatic lesions in vivo. Mechanistically, LINC01559 binds to vimentin and prevents its ubiquitination and proteasomal degradation, leading to promotion of LUAD cell migration, invasion, and metastasis.

**Conclusion:**

LINC01559 plays an important role in LUAD metastasis through stabilizing vimentin. The expression of LINC01559 is potentially an independent prognostic factor of LUAD patients, and LINC01559 targeting may represent a novel avenue for the treatment of late-stage LUAD.

**Supplementary Information:**

The online version contains supplementary material available at 10.1186/s40364-024-00571-3.

## Background

Lung cancer is one of the most frequently diagnosed cancers and the leading cause of cancer-related death worldwide [1–3], with lung adenocarcinoma (LUAD) representing the most common subtype of all diagnoses [4, 5]. Early-stage LUAD can be cured by radical resection, but there is currently no curative treatment for patients with late-stage LUAD, despite the recent progression in molecularly targeted therapy, immunotherapy, and combinatorial therapy [6–8]. This is closely associated with metastasis of the disease beyond the original site [9]. However, the molecular mechanisms involved in LUAD metastasis remain to be fully understood, although it is known that the epithelial-to-mesenchymal transition (EMT) is essential in driving the metastatic process [10–13], and that vimentin, a type of intermediate filament (IF) protein, plays a critical role in reducing cell-cell adhesion and reorganizing the cellular architecture to allow cells to become more migratory and invasive during the EMT [10, 14].

Almost all current cancer biomarkers and therapeutics target proteins and the genes that code for those proteins. Nevertheless, it is increasingly appreciated that non-coding RNAs (ncRNAs), including long ncRNAs (lncRNAs), play important roles in diverse pathobiological processes [15–17]. In particular, the dysregulation of lncRNAs are critically involved in the pathogenesis of cancer [18–20]. LncRNAs frequently function to establish intermolecular interactions with other biomolecules, including proteins, DNAs, and other RNAs [18, 21–24]. This enables lncRNAs to act as tethers, guides, decoys or scaffolds to undertake various biological functions, including cancer development, progression, and treatment resistance [25–28]. For example, the lncRNA pan-cancer lncRNA activating NCOR2 responsive to E2F1 (PLANE) regulates an alternative splicing program to promote cancer pathogenesis in the pan-cancer context [21], whereas the lncRNA glycoLINC (gLINC) acts as a backbone to assemble a glycolytic metabolon, thus promoting glycolysis to enable cancer cell adaptation to metabolic stress [29]. Of note, the list of dysregulated lncRNAs that contribute to LUAD pathogenesis is also expanding rapidly. For instance, while the lncRNA c-Myc-inducible long noncoding RNA inactivating p53 (MILIP) represses the tumor suppressor p53 and promotes tumorigenesis in diverse cancer types including LUAD [30], the lncRNA lysosome cell death regulator (LCDR) regulates the integrity of lysosomal membrane and promote LUAD cell survival [31]. Nevertheless, the role of lncRNAs in regulating LUAD metastasis remains to be fully elucidated.

Here, we demonstrate that the lncRNA LINC01559 (Long Intergenic Non-protein Coding RNA 1559) binds to and stabilizes vimentin through preventing its ubiquitination, and thus promotes the EMT and metastasis in LUAD cells. Moreover, we show that LINC01559 is frequently upregulated in metastatic LUADs and is an independent prognostic factor of LUAD patients, with practically relevant implications of LINC01559 targeting for counteracting LUAD metastasis.

## Methods

### Cell culture and human tissues

The human LUAD cell lines A549, H1299, H1975, and H838 were from Cell Bank of the Chinese Academy of Sciences. These cell lines were maintained in RPMI-1640 (Invitrogen, # 8123228; CA, USA) supplemented with 10% fetal bovine serum (FBS, Gibco, # A5669701; CA). The human skin fibroblast line CCC-HSF-1 from Cell Bank of the Chinese Academy of Sciences and the human embryonic kidney epithelial-like cell line 293 T from American Type Culture Collection (ATCC) were cultured in DMEM (Invitrogen, # 11965-118; CA) supplemented with 10% FBS and 1% penicillin-streptomycin. Cells were cultured in a humidified incubator at 37 °C and 5% CO_2_. All cell lines were verified to be free of mycoplasma contamination every 3 months. Formalin-fixed paraffin-embedded LUAD cancer tissue microarrays were purchased from the Shanghai Outdo Biotech Co., Ltd. (HLugA180Su11).

### Antibodies (Abs)

The mouse monoclonal antibody (mAb) against vimentin (Cat# sc-6260) was from Santa Cruz Biotechnology (Dallas, TX). The mouse mAb against ubiquitin (Cat# 3936S) was from Cell Signaling Technology (Danvers, MA). The mouse mAb against β-actin (Cat# A5441) was from Sigma-Aldrich (Missouri, USA). The goat-anti-mouse IgG(H + L)-HRP conjugate (Cat# A0216) and the non-specific mouse IgG (Cat# A7028) were from Beyotime Biotechnology (Beijing, China).

### Reagents

Cycloheximide (CHX; Cat# 66-81-9) was purchased from MedChemExpress (New Jersey, USA). Dimethyl sulfoxide (DMSO; Cat# 67-68-5), tris hydrochloride (Tris-HCl; Cat# 1185-53), magnesium chloride (MgCl_2_; Cat# 7786-30), polybrene (Cat# 28728-55-4) and Triton X-100 (Cat# 9036-19-5) were purchased from Sigma-Aldrich (Missouri, USA). MG132 (Cat# S2619) was purchased from Selleckchem (Houston, USA). RIPA Lysis Buffer (Cat# P0013B) and Phenylmethanesulfonyl fluorid (PMSF; Cat# ST505) were purchased from Beyotime Biotechnology (Beijing, China). Pierce™ BCA Protein Assay Kit (Cat# 23227) was purchased from ThermoFisher Scientific (MA, USA).

### SiRNAs and short hairpin RNA (shRNA) oligos

SiRNAs were obtained from Ribobio (Guangzhou, China) and transfected using the lipofectamine 3000 Transfection Kit (ThermoFisher Scientific, #L3000-015). ShRNA oligos were purchased from TSINGKE Biological Technology (Beijing, China). The siRNA sequences directed against LINC01559 were GTAGGTGACTACAGTTAAT (LINC01559 siRNA1) and GCAAGAAGCTGGAAATCGA (LINC01559 siRNA2). The siRNA sequences directed against vimentin were GCAGAAGAATGGTACAAAT (Vimentin siRNA1) and CAACGAAACTTCTCAGCAT (Vimentin siRNA2). The shRNA sequence targeting LINC01559 was GATTATTTATTGTCTACTTAT (LINC01559 shRNA).

### Plasmids

The pcDNA3.1 plasmid, pcDNA3.1-LINC01559 plasmid and pcDNA3.1-vimentin plasmid were purchased from TSINGKE Biological Technology (Beijing, China).

### Quantitative real-time PCR (qPCR)

Total RNA was extracted from cultured cells using the FastPure^®^ Cell/Tissue Total RNA Isolation Kit V2 (Vazyme, # RC112; Nanjing, China) according to the manufacturer’s instructions. 15 ng cDNA synthesized from 900 ng of total RNA using the PrimScript^TM^ RT reagent Kit with gDNA Eraser (TaKaRa, #RR047A; Dalian, China) was subjected to qPCR using a StepOnePlus™ Real-Time PCR System (ThermoFisher Scientific). 2^−-ΔΔCT^ method was used to calculate the relative gene expression levels normalized against β-actin. Primer sequences are listed in Supplementary Table 1.

### Wound healing assay

The culture-insert 2 Well for Self-Insertion (iBiDi, #190718/6, Germany) is placed on the surface of the 24-well plate and provides two cell culture chambers with walls 500 μm thick between the two inserts. Laying cells in two small chambers and removing the culture-inserts with clean sterile tweezers left cells. After unplugging the culture-inserts, the residual floating cells were gently washed with PBS, and fresh medium containing 2% serum was added for scratch experiment. The wound area was photographed and recorded in the same field of vision during a specific time. The wound width was quantified using the Vision Works software (Analytik Jena, Jena, Germany).

### Transwell migration assay

Cells were seeded to upper chambers of 24-well transwell inserts (8μm, Corning, #3422; 5 × 10^4^ cells/well) and maintained in serum-free medium. The bottom chambers were filled with medium supplemented with 10% FBS. After 16 h, cells grown in inserts were fixed with 4% paraformaldehyde (Servicebio, #G1101; Wuhan, China) for 30 min and then stained using crystal violet stain solution (Beyotime, #C0121; Beijing, China) for 8 min. The non-migrated cells on the upper surface of the membranes were removed by scrubbing with a cotton tipped swab. Cells that migrated to the lower surface of the membranes were further imaged using Fluorescence Microscope System (Olympus, IX73) prior to quantification with the ImageJ software.

### Transwell invasion assay

Cell invasion was assayed using inserts pre-coated with an appropriate amount of Matrigel (BD Biosciences, # 356234; Franklin Lakes, NJ) mixed with precooled medium at a ratio of 1:8. Cells were seeded to upper chambers of 24-well transwell inserts (8μm, Corning, #3422; 1 × 10^5^ cells/well) and maintained in serum-free medium. The subsequent experimental procedure was similar to that of the above-described transwell migration assay.

### In situ hybridization (ISH)

ISH assays were performed using the RNAscope® 2.5 HD Detection Reagent-BROWN (Advanced Cell Diagnostics, #322310; CA, USA) according to the manufacturer’s instructions. Briefly, FFPE LUAD tissue microarray (HLugA180Su11) purchased from the Shanghai Outdo Biotech Co., Ltd (China) was deparaffinized in xylene for 5 min at RT twice, followed by dehybridization in 100% alcohol. After being air-dried, the tissue sections were incubated with hydrogen peroxide for 10 min at RT and washed five times in the distilled water. Then the sections were heated in target retrieval reagent to 100 °C for 20 min, followed by being treated with proteinase K and incubated in hybridization buffer containing probes (Advanced Cell Diagnostics, #1042471-C1) at 40 °C for 5 h. After being washed, the sections were incubated with 3,3′-diaminobenzidine (DAB), and counterstaining was carried out using hematoxylin. The percentage of positive cells was ranged from 0 to 100%. The intensity of staining (intensity score) was judged on an arbitrary scale of 0 to 4: no staining (0), weakly positive staining (1), moderately positive staining (2), strongly positive staining (3) and very strong positive staining (4). A reactive score (RS) was derived by multiplying the percentage of positive cells with staining intensity divided by 10.

### RNA pull-down (RPD)

Cells were harvested and lysed in lysis buffer (50 mM Tris-HCl [pH 7.5], 150 mM NaCl, 2.5 mM MgCl_2_, 1 mM EDTA, 10% Glycerol, 0.5% Nonidet P-40/Igepal CA-630, 1 mM DTT, cOmplete™ EDTA-free Protease Inhibitor Cocktail and RNase inhibitors). 4 µg antisense/sense biotin-labelled probes were incubated with lysates at 4 °C overnight before rotating with streptavidin Agarose resin (ThermoFisher Scientific, #20349) for additional 2 h. Beads were then washed in lysis buffer for six times, followed by RNA isolation and immunoblotting analysis using Image Studio software. The sequences of LINC01559 AS probes were GCAGAAGAATGGTACAAAT (Antisense 1), TCGATTTCCAGCTTCTTGC (Antisense 2), ACTTATTCAAGGTCATGGC (Antisense 3) ATAAGTAGACAATAAATAATC (Antisense 4) and AATAATATACAATAAGTAGAC (Antisense 5). The sequences of LINC01559 S probes were GTAGGTGACTACAGTTAAT (Sense 1), GCAAGAAGCTGGAAATCGA (Sense 2), GCCATGACCTTGAATAAGT (Sense 3), GATTATTTATTGTCTACTTAT (Sense 4), and GTCTACTTATTGTATATTATT (Sense 5). Dilution of vimentin antibody was 1:1000.

### RNA immunoprecipitation (RIP)

A549 and H1299 cell pellets were lysed using RIP lysis buffer (KCl 150 mM, pH7.4 Tris-HCl 25 mM, EDTA 5 mM, Nonidet P-40 0.5%, Glycerol 20 mL, DTT 0.5 mM) supplemented with RNase and protease inhibitors and sonicated. 1 µg vimentin antibody or control IgG were incubated with lysates at 4 °C overnight after rotating with magnetic beads (ThermoFisher Scientific, #26162; MA, USA) for additional 30 min at 37 °C. After being washed with RIP wash buffer, the bead-bound immunocomplexes were subjected to immunoblotting analysis and RNA isolation.

### Mass spectrometry (MS) analysis

Proteins co-pulled down with RNA using antisense / sense biotin-labelled probes were separated by 10% acrylamide gels and visualized by Coomassie brilliant blue staining. The specific protein recovered using antisense probes were excised and digested, followed by liquid chromatography-mass spectrometry (LC-MS) analysis (ThermoFisher Scientific, EASY-nLC1000 & LTQ Orbitrap Velos Pro). Proteins identified from the mass spectrometry analysis are listed in Supplementary Table 2.

### Ubiquitination assay

Cell lysates were obtained by lysis buffer (SDS 2%, NaCl 150 mM, Tris-HCl pH8.0 10 mM, Phospholipase inhibitors 2 mM, NaF 50 mM), and immunoprecipitation was performed with vimentin antibody overnight at 4 °C. Magnetic beads (ThermoFisher Scientific, #26162; MA, USA) were then added to incubate overnight for 4 h at 4 °C. Finally, the proteins were analyzed using western blot assay using ubiquitin antibody (P4D1, CST, #3936S). Dilution of vimentin antibody was 3μg/ml buffer. Dilution of P4D1 antibody was 1:1000.

### LUAD metastasis mouse model

Twelve non-obese diabetic/Server combined immune-deficiency (NOD/SCID) mice (male, 4 weeks of age) were purchased from Shanghai Model Organisms (Shanghai, China) and housed in a temperature-controlled room (21-23 °C) with 40-60% humidity and a light/dark cycle of 12 h/12 h. Twelve mice were randomly divided into two groups. A549 cells with stable LINC01559 knockdown and control A549 cells diluted in 200ul PBS were injected through tail vein (3 × 10^6^ cells/mice). At the end of the 8^th^ week after tumor cell injection, mice were sacrificed the tumor nodules in both lungs of each mouse were qualified. LUAD metastasis was confirmed pathologically using H & E staining.

### Statistics

Statistical analysis was carried out using SPSS 19.0 (SPSS Software, Chicago, USA) or GraphPad Prism 9 to assess differences between experimental groups. Statistical differences were analyzed by two-tailed Student’s *t*-test or one-way ANOVA test followed by Tukey’s multiple comparisons. *P* values lower than 0.05 were considered to be statistically significant.

## Results

### LINC01559 upregulation is associated with LUAD metastasis and poor patient prognosis

**Fig. 1:**
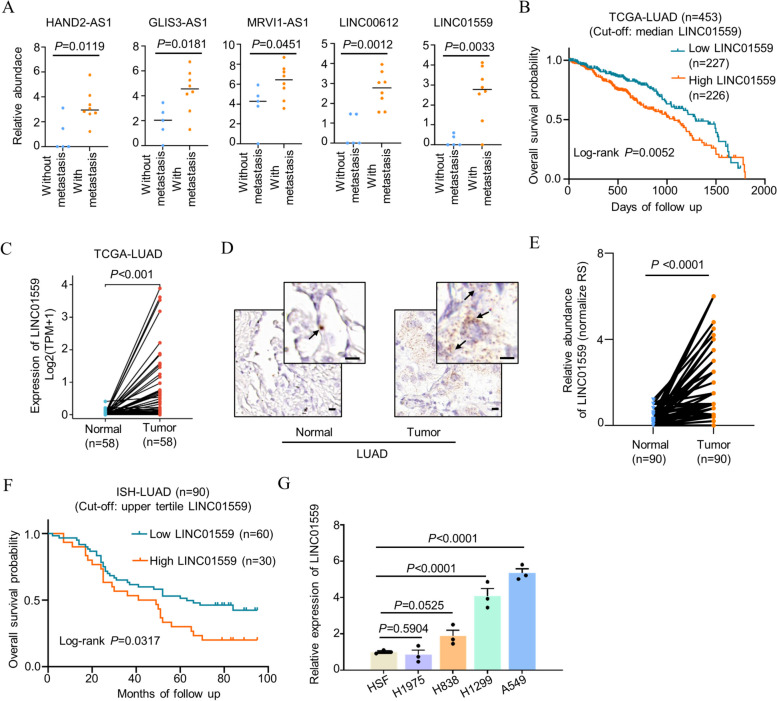
LINC01559 is expressed at higher levels in LUADs with metastasis and is associated with poor patient outcome. **A** Analysis of a published gene expression microarray dataset for treatment-naïve NSCLC (GSE11117) showing that the lncRNAs HAND2-AS1, GLIS3-AS1, MRVI1-AS1, LINC00612, and LINC01559 were expressed at higher levels in bronchoscopic biopsy tissues from LUADs with metastatic lesions compared to those without. Two-tailed Student’s *t*-test. **B** Kaplan-Meier analysis of the probability of overall survival of LUAD patients (*n* = 453) derived from the TCGA datasets. The log-rank test. **C** LINC01559 was expressed at higher levels in LUAD tissues (*n* = 58) compared to paired adjacent normal tissues (*n* = 58) in the TCGA database. Two-tailed Student’s *t*-test. **D** and **E** Representative microphotographs (**D**) and quantitation (**E**) of in situ hybridization (ISH) analysis of LINC01559 expression in formalin-fixed paraffin-embedded (FFPE) lung adenocarcinoma (*n* = 90 biologically independent samples) compared with paired adjacent normal tissues. Scale bar, 10 µm. RS: reactive score. Two-tailed Student’s *t*-test. **F** Kaplan-Meier analysis of the probability of overall survival of LUAD patients (*n* = 90) stratified according to the expression of LINC01559 as shown in **D** and **E**. The log-rank test. **G** qPCR analysis of LINC01559 expression in the indicated LUAD cell lines and the human fibroblast cell line HSF. Data are mean ± s.d.; *n* = 3 independent experiments, one-way ANOVA followed by Tukey’s multiple comparisons test

To better understand the role of lncRNAs in LUAD metastasis, we interrogated a previously published gene expression microarray dataset for treatment-naïve non-small cell lung carcinoma (NSCLC) included in the Gene Expression Omnibus (GEO) repository (GSE11117), which comprises LUAD cases of different pathological stages [32]. This analysis revealed that 5 annotated lncRNAs (HAND2-AS1, GLIS3-AS1, MRVI1-AS1, LINC00612, and LINC01559) were expressed at higher levels in bronchoscopic biopsy tissues from LUADs with metastatic lesions compared to those without (Fig. 1A). Intriguingly, high expression of LINC01559, but not the other lncRNAs, was associated with LUAD patient overall survival (OS), as shown by analysis of the LUAD dataset in the cancer genome atlas (TCGA) (Fig. 1B). Indeed, LINC01559 was expressed at increased levels in LUAD compared with paired non-cancerous lung tissues in the TCGA LUAD dataset (Fig. 1C).

We confirmed the upregulation of LINC01559 in 90 formalin-fixed paraffin-embedded (FFPE) LUAD samples compared to paired noncancerous lung tissues using in situ hybridization analysis (Fig. 1D and E, and Table 1). There were no significant differences in LINC01559 expression in LUAD of different groups stratified by patient gender as well as their median age at diagnosis (Table 1). However, high LINC01559 expression appeared to be associated with higher histological grade (grade III-IV) and late-stage [American joint committee on cancer (AJCC) stage III-IV] of LUAD (Table 1). Further analysis revealed that high LINC01559 expression was associated with poor patient OS (Fig. 1F). Furthermore, multivariable Cox regression analysis revealed that high LINC01559 expression was associated with OS independently of stage and age at diagnosis, well-established prognostic markers of LUAD patients (Table 2). Similarly, LINC01559 was expressed at higher levels in 3 of 4 LUAD cell lines than the human fibroblast cell line HSF (Fig. 1G). Taken together, these results indicated that LINC01559 is upregulated in LUAD, in particular, in those with metastasis, and its high expression is associated with poor prognosis of LUAD patients. We therefore focused on investigation of the potential role of LINC01559 in regulating LUAD metastasis and molecular mechanisms involved.

**Table 1:** Clinicopathological characteristics of the cohort of 90 LUAD patients

Characteristics	LINC01559 expression		*P* Value^a^
	Low	High	
Sex			
Male	23	25	0.673
Female	22	20	
Age			
≤ 60	23	22	0.833
> 60	22	23	
Histological grade			
Grade I-II	37	27	0.020
Grade III-IV	8	18	
Tumor location			
Left lung	16	14	0.655
Right lung	29	31	
AJCC^b^ Stage			
Stage I-II	38	29	0.030
Stage III-IV	7	16	
T stage			
T1-2	40	41	1.000
T3-4	5	4	

^a^Chi-square test; a *P* value less than 0.05 was considered statistically significant

^b^*AJCC* American Joint Committee on Cancer

**Table 2:** Multivariable Cox regression analysis of prognostic factors of outcome in 90 LUAD patients

Factors	Overall survival	
	HR^b^ (95%CI)	*P* value
High LINC01559 expression (upper tertile as the cut off)^a^	2.193 (1.272–3.781)	0.005
Stages III-IV^c^	3.127 (1.699–5.755)	0.0002
Age > 60 years	1.659 (0.983–2.801)	0.058

^a^LINC01559 expression levels were considered high or low in relation to the upper tertile level of expression in all tumors analyzed

^b^Hazard ratios were calculated as the antilogs of the regression coefficients in the proportional hazards regression. Multivariable Cox regression analysis was performed following the inclusion of the three above-listed factors into the Cox regression model

^c^Tumor stage was categorized as favorable (American Joint Committee on Cancer stages I-II) or unfavorable (American Joint Committee on Cancer stages III-IV)

### LINC01559 promotes LUAD metastasis

**Fig. 2:**
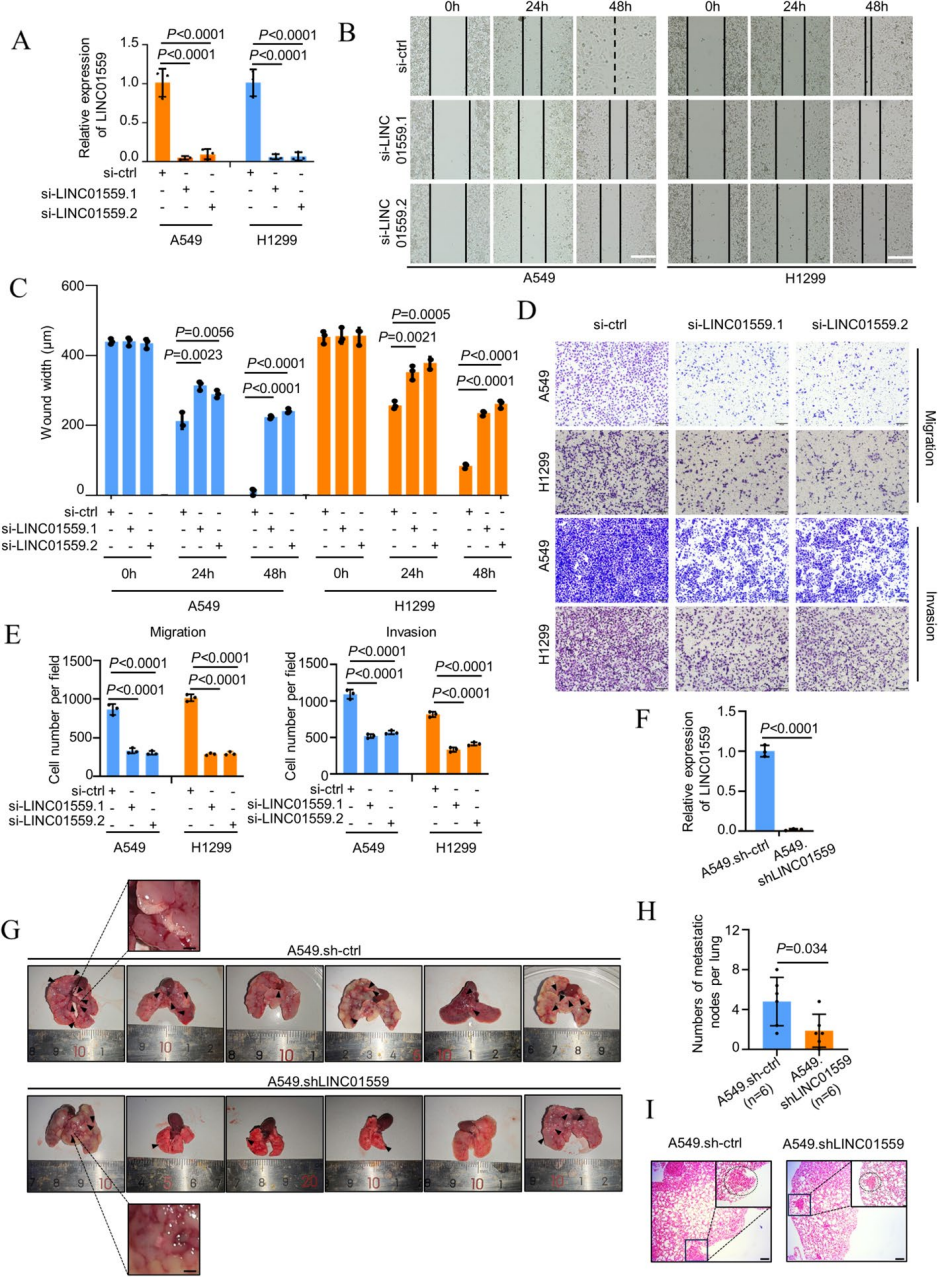
LINC01559 promotes LUAD metastasis. **A**-**E** SiRNA knockdown of LINC01559 (**A**) inhibited wound healing (**B**, **C**) and migration and invasion **(D**, **E**) of A549 and H1299 cells. Wound width and the numbers of migrated and invasive cells were quantitated using the ImageJ software. Scale bars, 200 μm (wound healing assay) and 200 μm (cell migration and invasion assay). Data are mean ± s.d.; *n* = 3 independent experiments, one-way ANOVA followed by Tukey’s multiple comparisons test. **F** and **H** Stable knockdown of LINC01559 using shRNA (**F**) inhibited A549 cell lung metastasis in vivo (**G**). Scale bars, 0.1 cm. The number of metastatic lesions was quantified (**H**) by counting lung nodules from lungs harvested from NOD/SCID mice that received tail vein injection of A549.sh-ctrl or A549.shLINC01559 cells. *n* = 6 biologically independent animals; Two-tailed Student’s *t*-test. **I** Representative photographs of Hematoxylin and Eosin (H&E) staining of lung tissues from NOD/SCID that received tail vein injection of A549.sh-ctrl or A549.shLINC01559 cells. Scale bar, 100 μm. Data are representatives of six biologically independent animals

To functionally examine the role of LINC01559 in regulating LUAD metastasis, we knocked down LINC01559 in A549 and H1299 cells using two independent siRNAs (Fig. 2A). Indeed, siRNA knockdown markedly delayed wound healing of the LUAD cells in scratch assays and reduced their migration and invasion into Matrigel in transwell assays (Fig. 2B-E). In contrast, overexpression of LINC01559 promoted A549 and H1299 cell wound healing, migration, and invasion (Supplementary Fig. 1A-E).

To facilitate further investigations, we established the A549 subline, A549.shLINC01559 with LINC01559 stably knocked down using shRNA (Fig. 2F). Similar to siRNA knockdown, shRNA knockdown of LINC01559 triggered reductions in cell migration and invasion (Supplementary Fig. 1F). Moreover, NOD/SCID mice that received tail vein injection of A549. shLINC01559 cells exhibited marked reductions in the formation of lung metastatic lesions compared with those that were injected with A549 carrying the control shRNA (Fig. 2G and H). In accordance, the average weight of lungs harvested from mice that received vein injection of A549.shLINC01559 cells was significantly reduced relative to the controls (Supplementary Fig. 1G). The pathological confirmation of lung metastasis was performed using hematoxylin and eosin (H & E) staining in randomly selected lung nodules (Fig. 2I). Collectively, these results indicate that LINC01559 promotes LUAD metastasis in vivo.

### LINC01559 interacts with vimentin

**Fig. 3:**
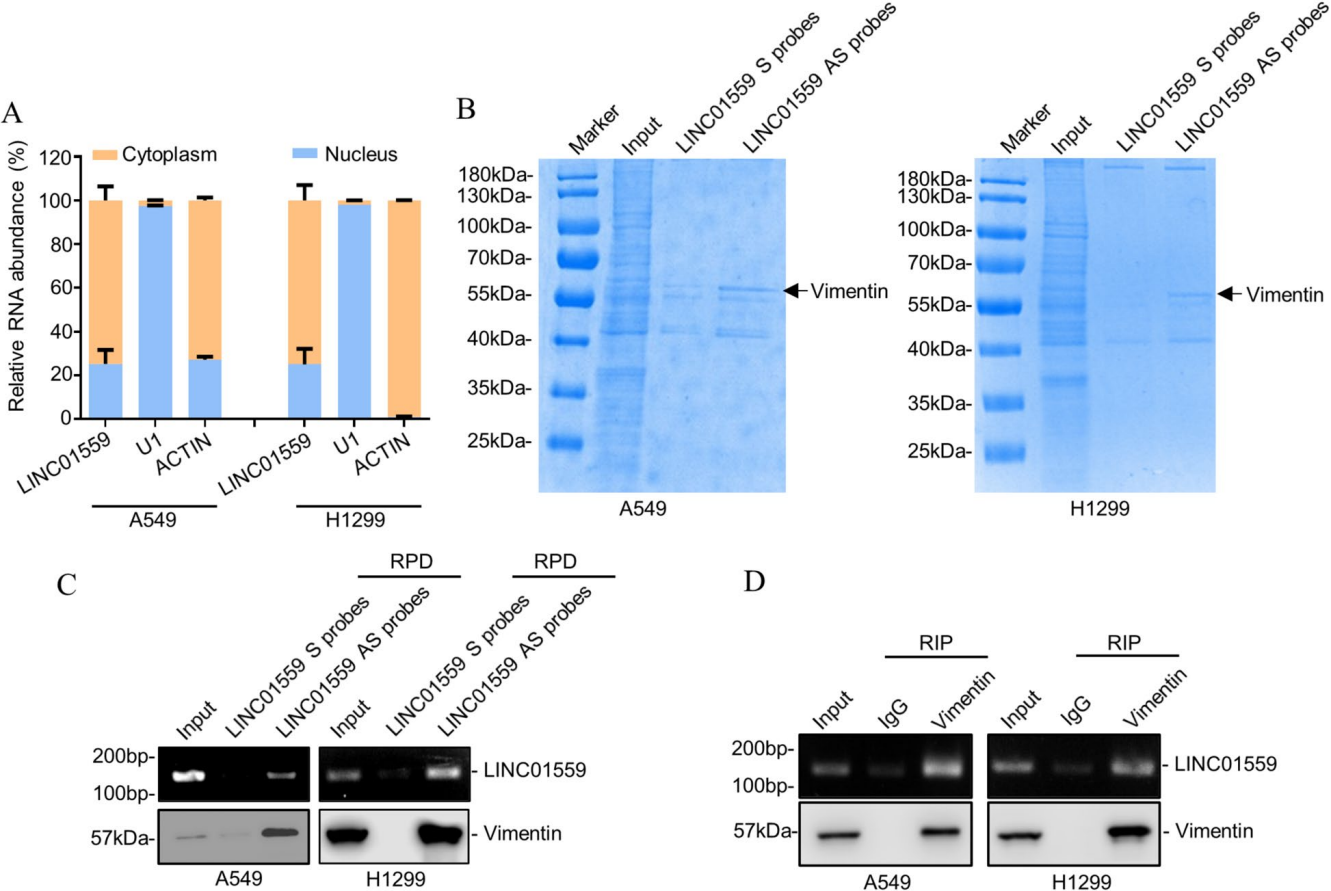
LINC01559 binds to vimentin. **A** qPCR analysis showing LINC01559 is predominantly located to the cytoplasm. Analysis of β-actin was included as a cytoplasmic marker, and U1, a nuclear marker. Data are mean ± s.d.; *n* = 3 independent experiments. **B** RNA pulldown followed by mass spectrometry analysis identified that vimentin was the most abundant protein co-pulled down with LINC01559. S: sense; AS: antisense. *n* = 1 experiment. **C** Vimentin was co-pulled down with LINC01559 in A549 and H1299 cells as shown in RNA pulldown assays. RPD, RNA pulldown. *n* = 3 independent experiments. **D** LINC01559 was co-precipitated with vimentin in A549 and H1299 cells as shown in RNA immunoprecipitation assays. RIP: RNA immunoprecipitation. *n* = 3 independent experiments

To understand the mechanism responsible for LINC01559-mediated promotion of LUAD metastasis, we examined its subcellular localization using qPCR analysis of subcellular fractions in A549 and H1299 cells. This analysis demonstrated that LINC01559 was predominantly located to the cytoplasm in LUAD cells (Fig. 3A). Strikingly, when we analyzed the proteins that interact with LINC01559 using RNA pulldown (RPD) followed by mass spectrometry, it appeared the most abundant protein that coprecipitated with LINC01559 was vimentin (Fig. 3B and Supplementary Table 2), an intermediate filament protein that is primarily located to the cytoplasm and is closely associated with cell motility, invasiveness, and metastasis [33, 34]. We subsequently confirmed the association of LINC01559 and vimentin using RPD and RNA immunoprecipitation (RIP) assays in A549 and H1299 cells (Fig. 3C and D). Together, these results suggest that the interaction between LINC01559 and vimentin may play a role in LINC01559-mediated promotion of LUAD cell metastasis.

### Vimentin is necessary for LINC01559-mediated LUAD cell migration and invasion

**Fig. 4:**
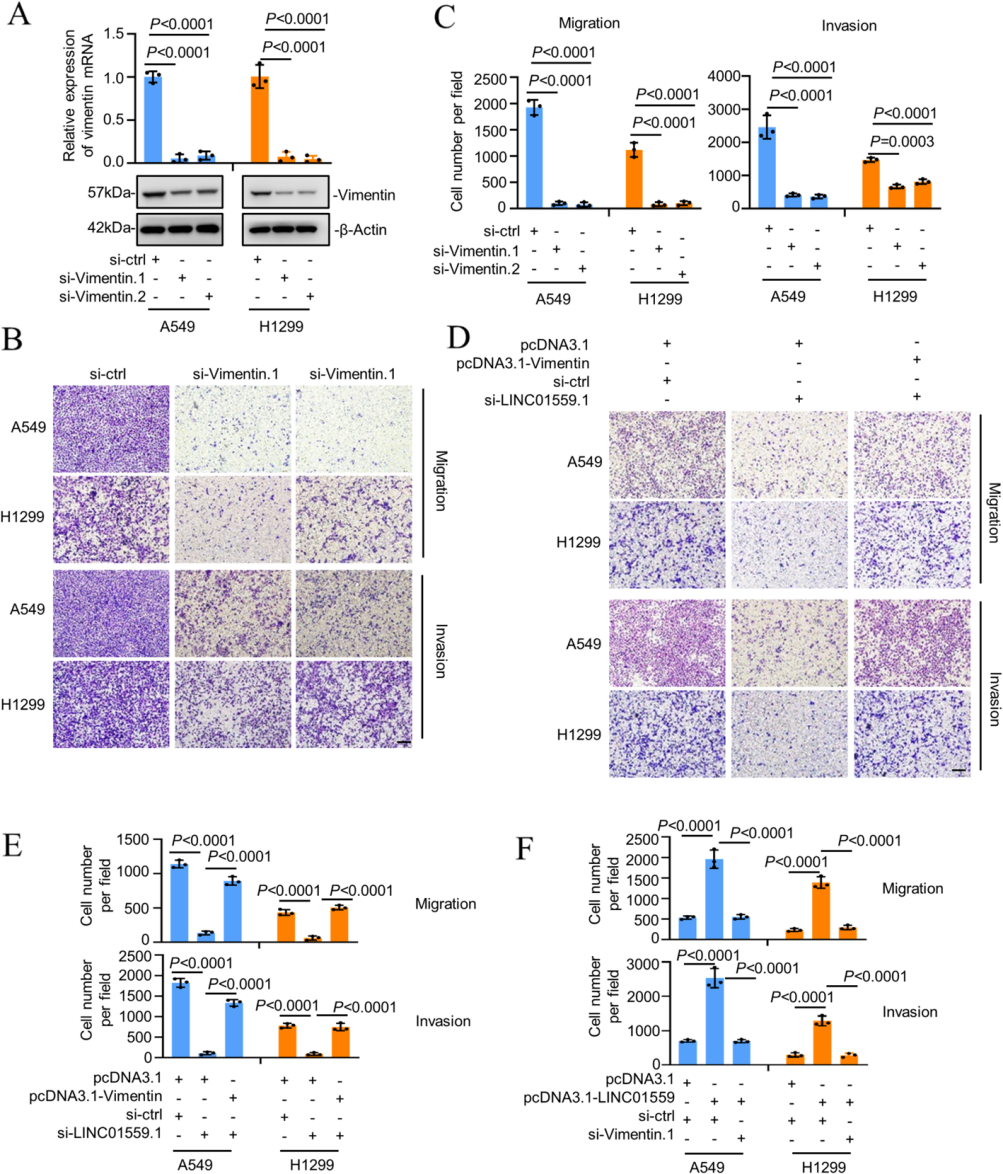
Vimentin is necessary for LINC01559-mediated LUAD cell migration and invasion. **A**-**C** SiRNA knockdown of vimentin (**A**) reduced migration and invasion (**B**, **C**) of A549 and H1299 cells. Scale bars 200 μm. The numbers of migrated and invasive cells were quantitated using the ImageJ software. Data are mean ± s.d. (A and C); *n* = 3 independent experiments, one-way ANOVA followed by Tukey’s multiple comparisons test. **D** and **E** Overexpression of vimentin prevented the reductions in migration and invasion caused by siRNA knockdown of LINC01559 in A549 and H1299 cells. The numbers of migrated and invasive cells were quantitated using the ImageJ software. Scale bars 200μm. Data are mean ± s.d. (E); *n* = 3 independent experiments, one-way ANOVA followed by Tukey’s multiple comparisons test. **F** SiRNA knockdown of vimentin diminished the increases of cell migration and invasion caused by LINC01559 overexpression in A549 and H1299 cells. Scale bars 200μm. Data are mean ± s.d.; *n* = 3 independent experiments, one-way ANOVA followed by Tukey’s multiple comparisons test

We next investigated the functional importance of vimentin in LINC01559-mediated LUAD cell migration and invasion. As anticipated [35], vimentin was markedly upregulated in LUAD cells (Supplementary Fig. 2A). Moreover, siRNA knockdown of vimentin diminished A549 and H1299 cell invasion and migration, as shown in transwell assays (Fig. 4A-C). In contrast, overexpression of vimentin promoted invasion and migration of LUAD cells (Supplementary Fig. 2B-D). Importantly, further analysis showed that vimentin overexpression prevented the reductions in migration and invasion caused by siRNA knockdown of LINC01559, whereas knockdown of vimentin inhibited the promotion of migration and invasion resulted from LINC01559 overexpression (Fig. 4D-F). Thus, vimentin is required for LINC01559-mediated promotion of LUAD cell migration and invasion.

### LINC01559 promotes vimentin expression

**Fig. 5:**
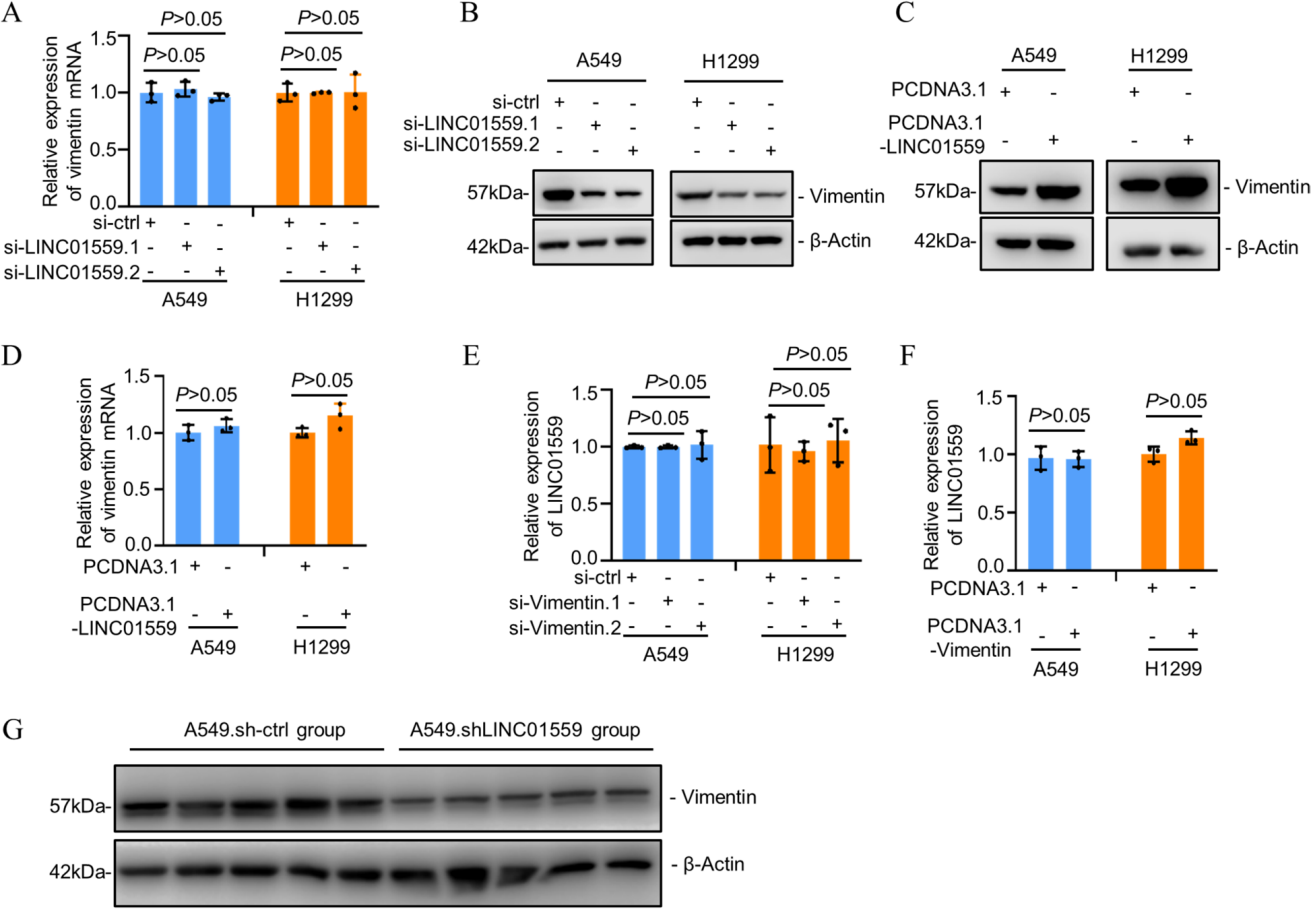
LINC01559 promotes vimentin expression. **A** SiRNA knockdown of LINC01559 did not affect the levels of vimentin mRNA in A549 and H1299 cells. Data are mean ± s.d.; *n* = 3 independent experiments, one-way ANOVA followed by Tukey’s multiple comparisons test. **B** SiRNA knockdown of LINC01559 reduced vimentin protein expression in A549 and H1299 cells. *n* = 3 independent experiments. **C** and **D** Overexpression of LINC01559 upregulated vimentin at the protein level (C), but did not alter vimentin mRNA expression (D). Data are mean ± s.d. (D); *n* = 3 independent experiments, one-way ANOVA followed by Tukey’s multiple comparisons test. **E** and **F** SiRNA knockdown or overexpression of vimentin did not cause any significant changes in the levels of LINC01559 expression in in A549 and H1299 cells. Data are mean ± s.d.; *n* = 3 independent experiments, one-way ANOVA followed by Tukey’s multiple comparisons test. **G** A549.shLINC01559 lung metastatic tumors exhibited reduced levels of the vimentin protein compared with lung metastatic tumors formed by A549.sh-ctrl cells. *n* = 5 biologically independent lung metastatic tumors

To further understand the significance of the interaction between LINC01559 and vimentin, we tested the expression of vimentin in A549 and H1299 cells with or without LINC01559 knockdown. Intriguingly, although siRNA knockdown of LINC01559 did not cause any significant changes in the levels of vimentin mRNA as measured using qPCR (Fig. 5A), it markedly reduced vimentin protein expression in A549 and H1299 cells (Fig. 5B). In contrast, overexpression of LINC01559 resulted in upregulation of vimentin at the protein level (Fig. 5C), whereas it did not lead to any noticeable changes in vimentin mRNA expression (Fig. 5D). On the other hand, neither knockdown nor overexpression of vimentin triggered alterations in the expression of LINC01559 in A549 and H1299 cells (Fig. 5E and F). Therefore, LINC01559 promotes vimentin expression through a post-transcriptional increase. Instructively, LUAD cells harvested from A549.shLINC01559 lung metastasis similarly exhibited lower vimentin protein levels compared to those derived from A549 cells carrying the control shRNA (Fig. 5G), indicating that LINC01559 regulates vimentin expression in LUAD cells in vivo.

### LINC01559 stabilizes vimentin through inhibiting its ubiquitination

**Fig. 6:**
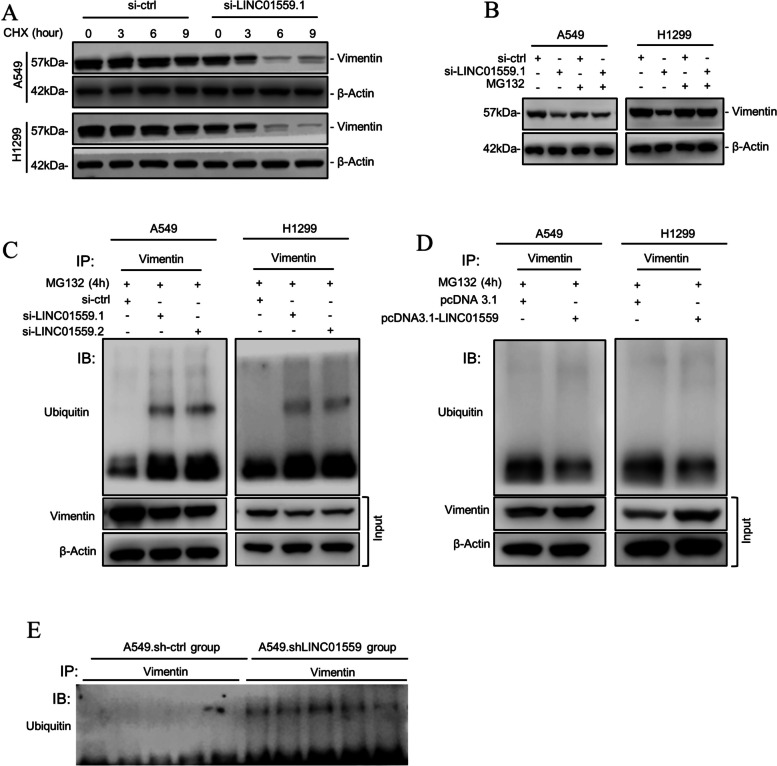
LINC01559 stabilizes vimentin. **A** Knockdown of LINC01559 accelerated the turnover of the vimentin protein in A549 and H1299 cells as shown in cycloheximide (CHX)-chase assays. *n* = 3 independent experiments. **B** Treatment with MG132 diminished the reductions in the vimentin protein caused by LINC01559 knockdown in A549 and H1299 cells. *n* = 3 independent experiments. **C** and **D** SiRNA knockdown of LINC01559 increased (**C**), whereas LINC01559 overexpression decreased (**D**) vimentin polyubiquitination in A549 and H1299 cells. *n* = 3 independent experiments. **E** A549.shLINC01559 lung metastatic tumors exhibited stronger vimentin polyubiquitination compared with lung metastatic tumors formed by A549.sh-ctrl cells. *n* = 5 biologically independent lung metastatic tumors

Having found that LINC01559 promotes vimentin expression through post-transcriptional upregulation, we carried out cycloheximide (CHX)-chase assays in A549 and H1299 cells with or without LINC01559 knockdown. Indeed, siRNA knockdown of LINC01559 accelerated the turnover of the vimentin protein (Fig. 6A), indicating that LINC01559 regulates the stability of vimentin. In support, treatment with the proteasome inhibitor MG132 diminished the reduction in vimentin caused by siRNA knockdown of LINC01559 (Fig. 6B). As polyubiquitination is required for proteasome-mediated protein degradation, we tested whether LINC01559 impinges on vimentin polyubiquitination. Indeed, siRNA knockdown of LINC01559 led to increases in vimentin polyubiquitination in A549 and H1299 cells (Fig. 6C). In contrast, overexpression of LINC01559 caused decreases in the polyubiquitination of vimentin in the cells (Fig. 6D). Importantly, vimentin polyubiquitination was stronger in LUAD cells harvested from A549.shLINC01559 lung metastatic tumors, in comparison with those derived from A549.sh-ctrl lung metastasis (Fig. 6E). Taken together, these results suggest that LINC01559 promotes vimentin expression through inhibiting its polyubiquitination in LUAD cells.

## Discussion

Distant metastasis is the leading cause of LUAD-associated death [36]. Nevertheless, despite the recent progression in molecularly targeted therapy, immunotherapy and combination therapy, curative treatment of metastatic LUAD remains an unmet medical need [6–8]. A better understanding of molecular mechanisms that drive LUAD cell migration, invasion, and metastasis is urgently needed. In this report, we presented evidence that the lncRNA LINC01559 is commonly upregulated in metastatic LUADs and its high expression is associated with poor outcome of LUAD patients and is potentially an independent prognostic factor. Mechanistic investigations revealed that LINC01559 binds to and stabilizes vimentin, thus promoting LUAD cell migration and invasion. Our results bear practical implications of targeting LINC01559 as an avenue for preventing LUAD metastasis.

As an intergenic lncRNA, LINC01559 is frequently upregulated in cancer cells and has revealed itself to be intimately entwined with the pathogenesis of diverse types of cancers [37–42]. In particular, several previous studies have shown its role in regulating cancer metastasis [39, 41, 43]. These are represented by the findings that LINC01559 expedites gastric cancer cell migration and metastasis through recruiting Insulin-like growth factor 2 mRNA-binding protein 2 (IGF2BP2) to stabilize zinc finger E-box binding homeobox 1 (ZEB1) expression [40], whereas it promotes pancreatic cancer cell migration through the yes-associated protein (YAP)-mediated pathway [39]. Moreover, LINC01559 has been shown to function as a competing endogenous RNA (ceRNA) of miR-1343-3p to accelerate triple-negative breast cancer progression, and similarly as a ceRNA to promote the progression of colorectal cancer and pancreatic cancer [38, 43, 44]. Our results demonstrating that LINC01559 promotes LUAD cell migration, invasion, and metastasis not only shed new lights on the molecular mechanisms responsible for regulating the progression of LUAD, but also uncovered a novel means through which LINC01559 contributes to cancer pathogenesis. Although LINC01559-mediated mechanisms vary widely among different cancer types, it is consistent with the well-established notion that lncRNAs function in a highly cell- and tissue-dependent manner [45, 46].

LINC01559 is known to be transcriptionally upregulated by the transcription factor zinc finger E-box binding homeobox 1 (ZEB1) in gastric cancer [40]. Moreover, m6A RNA methylation of LINC01559 has also been reported to play a role in regulating its expression in colorectal cancer [37]. However, further studies are clearly needed to test whether these mechanisms are involved in the regulation of LINC01559 expression in LUAD cells. Similarly, while LINC01559 has been reported to be transferred from mesenchymal stem cells (MSCs) into gastric cancer [47], whether this also occurs in the context of other types of cancers, including LUAD remains to be clarified.

As an intermediate filament protein, vimentin is primarily expressed in mesenchymal cells, regulating cell shape, adhesion, and migration through its interactions with other proteins and its role in maintaining cytoskeletal structure, under physiological conditions [33, 34, 48–50]. Nevertheless, as we shown in LUAD cells in this study, vimentin expression is often upregulated in cancer cells, in particular, in those that have undergone the EMT [35]. While the EMT is a dynamic biological process where epithelial cells lose their characteristics, such as cell-cell adhesion and polarity, and gain mesenchymal traits, such as enhanced migratory capacity and invasiveness, which are essential for embryogenesis, wound healing, and cancer progression, the increased expression of vimentin has a crucial role in the execution of the EMT, particularly through facilitating the morphologic and functional changes of cells that allow them to become more motile and invasive [10, 51]. Vimentin therefore serves a mesenchymal marker during the EMT process in cancer cells [33, 34]. Interestingly, the *VIM* gene locus, which gives rise to vimentin, also transcribes the lncRNA VIM-antisense 1 (VIM-AS1) [52], whereas the expression of vimentin is subjected to the regulation by the lncRNA AGAP2-antisense RNA 1 (AGAP2-AS1) [52]. Furthermore, the lncRNA LINC00675 regulates phosphorylation of vimentin on Ser83 and thus impinges on gastric cancer progression [53]. Our finding that LINC01559 promotes vimentin expression through impeding its polyubiquintination adds an additional layer of the complexity of the interaction between vimentin and lncRNAs, reinforcing the importance of lncRNAs in controlling the expression and function of vimentin, and thus in regulating cancer metastasis.

**Fig. 7:**
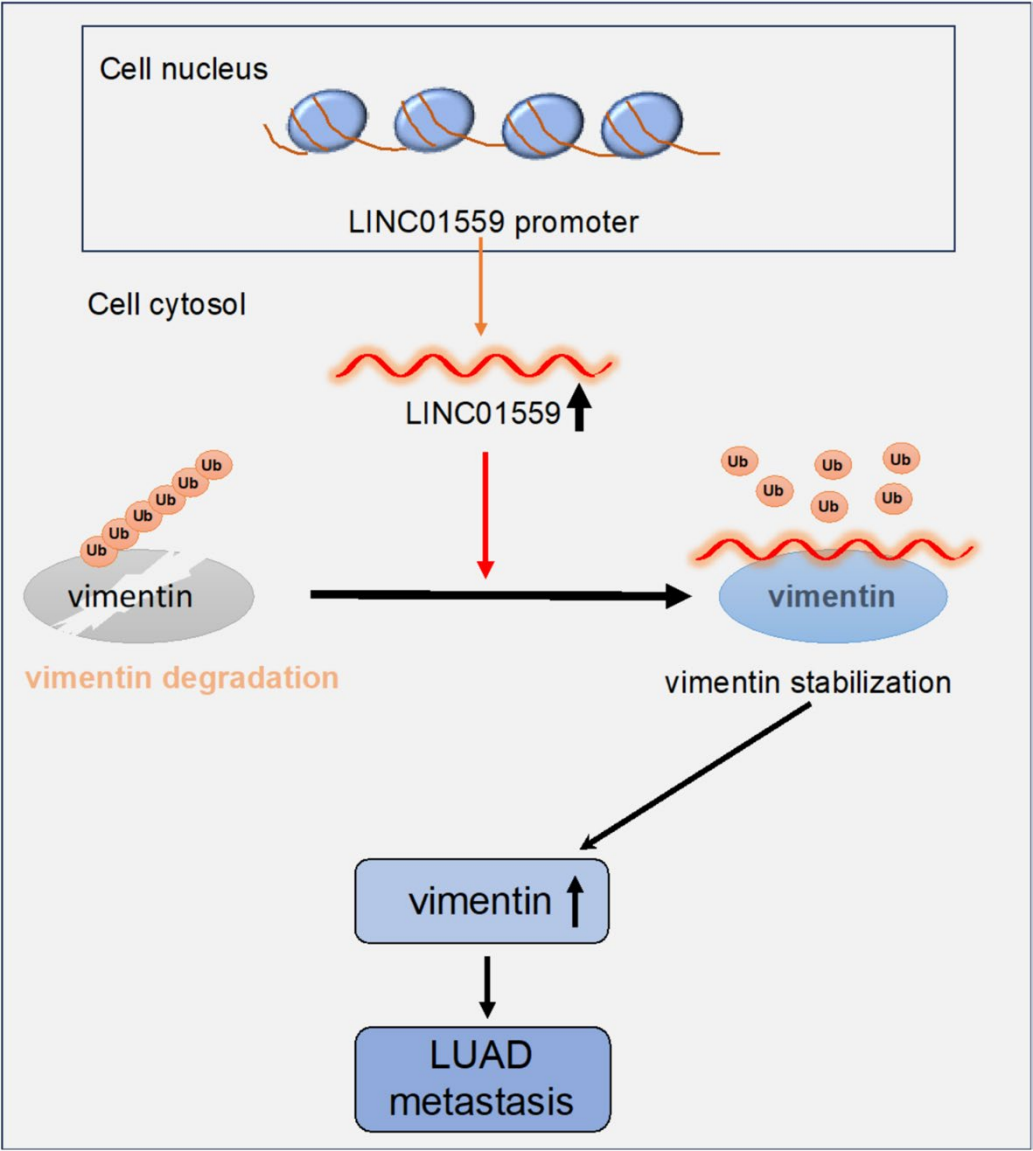
A schematic model of how LINC01559 promotes LUAD metastasis. LINC01559 binds to and stabilizes vimentin through preventing its ubiquitination, thus promoting LUAD metastasis

A pitfall of this study is that we were not able to apply our findings to spontaneous metastatic mouse models of LUAD that would functionally validate the role of LINC01559-mediated mechanism in LUAD metastasis in vivo. This is because that there is a lack of similarities between human LINC01559 and transcripts of Mus musculus that makes this approach impossible. Similarly, whether LINC01559 regulates vimentin ubiquitination in other types of cancers and whether LINC01559 regulate ubiquitination of other proteins remain open questions. Regardless, the results presented here identify a signal axis encompassing LINC01559 and vimentin that regulates LUAD metastasis and propose that LINC01559 may represent a molecular target for treatment of late-stage LUAD (Fig. 7). The practical potentials of LINC01559 are supported by the results from clinical samples, where LINC01559 expression is upregulated in metastatic LUAD tissues and is associated with poorer prognosis of LUAD patients.

## Conclusion

The lncRNA LINC01559 binds to vimentin and prevents its ubiquitination and proteasomal degradation, thus promoting LUAD cell migration, invasion, and metastasis. LINC01559 is upregulated in metastatic LUADs and high LINC01559 expression is associated with a poor prognosis of LUAD patients. These results unveil a novel lncRNA-mediated mechanism contributing to LUAD progression, and implicate that the expression of LINC01559 is likely an independent prognostic factor for LUAD patients, and that targeting LINC01559 represents a potential approach for the treatment of late-stage LUAD.

### Supplementary Information

**Additional file 1: Supplementary Figure 1.** Overexpression of LINC01559 promotes LUAD metastasis.

**Additional file 2: Supplementary Figure 2.** Overexpression of vimentin promotes LUAD metastasis.

**Additional file 3: Supplementary Table 1.** Primer sequences of quantitative real-time PCR.

**Additional file 4: Supplementary Table 2.** Summary of proteins that interact with LINC01559 in A549 cells detected using mass spectrometry.

**Additional file 5.**

## Data Availability

The datasets used and/or analyzed during the current study are available from the corresponding author S.C. on reasonable request.

## Abbreviations

CHX: Cycloheximide
DAB: Diaminobenzidine
EMT: Eepithelial-mesenchymal transition
H&E: Hematoxylin and eosin
IF: Intermediate filament
ISH: In situ hybridization
LUAD: Lung adenocarcinoma
lncRNAs: Long non-coding RNAs
NOD/SCID: Non-obese diabetic/server combined immune-deficiency
qPCR: Quantitative real‐time PCR
RIP: RNA immunoprecipitation
RPD: RNA pull-down
RS: Reactive score
shRNA: Short hairpin RNA
siRNA: Small interfering RNA

## References

[1] Thai AA, Solomon BJ, Sequist LV, Gainor JF, Heist RS (2021). Lung cancer. Lancet.

[2] Zhang T, Xia W, Song X, Mao Q, Huang X, Chen B (2022). Super-enhancer hijacking LINC01977 promotes malignancy of early-stage lung adenocarcinoma addicted to the canonical TGF-beta/SMAD3 pathway. J Hematol Oncol.

[3] Gao Q, Yang L, Lu M, Jin R, Ye H, Ma T (2023). The artificial intelligence and machine learning in lung cancer immunotherapy. J Hematol Oncol.

[4] Li H, Sha X, Wang W, Huang Z, Zhang P, Liu L (2023). Identification of lysosomal genes associated with prognosis in lung adenocarcinoma. Transl Lung Cancer Res.

[5] Zhang L, Yang Y, Lin W, Shao F, Gao Y, He J (2023). Complement-related molecular classification and a gene signature for lung adenocarcinoma. Exp Hematol Oncol.

[6] Liang J, Li H, Han J, Jiang J, Wang J, Li Y (2020). Mex3a interacts with LAMA2 to promote lung adenocarcinoma metastasis via PI3K/AKT pathway. Cell Death Dis.

[7] Yue D, Liu W, Gao L, Zhang L, Wang T, Xiao S (2021). Integrated multiomics analyses revealing different molecular profiles between early- and late-stage lung adenocarcinoma. Front Oncol.

[8] Yu XY, Wang M, Qian JJ (2022). CMaf-inducing protein promotes LUAD proliferation and metastasis by activating the MAPK/ERK pathway. Evid Based Complement Alternat Med.

[9] Zhang J, Fujimoto J, Zhang J, Wedge DC, Song X, Zhang J (2014). Intratumor heterogeneity in localized lung adenocarcinomas delineated by multiregion sequencing. Science.

[10] Manfioletti G, Fedele M (2023). Epithelial-Mesenchymal Transition (EMT). Int J Mol Sci.

[11] Zhou F, Sun J, Ye L, Jiang T, Li W, Su C (2023). Fibronectin promotes tumor angiogenesis and progression of non-small-cell lung cancer by elevating WISP3 expression via FAK/MAPK/ HIF-1alpha axis and activating wnt signaling pathway. Exp Hematol Oncol.

[12] Huang S, Gong N, Li J, Hong M, Li L, Zhang L (2022). The role of ncRNAs in neuroblastoma: mechanisms, biomarkers and therapeutic targets. Biomark Res.

[13] Huang Y, Hong W, Wei X (2022). The molecular mechanisms and therapeutic strategies of EMT in tumor progression and metastasis. J Hematol Oncol.

[14] Dong B, Li S, Zhu S, Yi M, Luo S, Wu K (2021). MiRNA-mediated EMT and CSCs in cancer chemoresistance. Exp Hematol Oncol.

[15] Aronoff GR, Bergstrom RF, Bopp RJ, Sloan RS, Callaghan JT (1988). Nizatidine disposition in subjects with normal and impaired renal function. Clin Pharmacol Ther.

[16] Liu Y, Shi M, He X, Cao Y, Liu P, Li F (2022). LncRNA-PACERR induces pro-tumour macrophages via interacting with miR-671-3p and m6A-reader IGF2BP2 in pancreatic ductal adenocarcinoma. J Hematol Oncol.

[17] Zhao R, Fu J, Zhu L, Chen Y, Liu B (2022). Designing strategies of small-molecule compounds for modulating non-coding RNAs in cancer therapy. J Hematol Oncol.

[18] Yu JE, Ju JA, Musacchio N, Mathias TJ, Vitolo MI (2020). Long noncoding RNA DANCR activates Wnt/beta-catenin signaling through MiR-216a inhibition in non-small cell lung cancer. Biomolecules.

[19] George J, Patel T (2015). Noncoding RNA as therapeutic targets for hepatocellular carcinoma. Semin Liver Dis.

[20] Xiao Y, Tang J, Yang D, Zhang B, Wu J, Wu Z (2022). Long noncoding RNA LIPH-4 promotes esophageal squamous cell carcinoma progression by regulating the miR-216b/IGF2BP2 axis. Biomark Res.

[21] Teng L, Feng YC, Guo ST, Wang PL, Qi TF, Yue YM (2021). The pan-cancer lncRNA PLANE regulates an alternative splicing program to promote cancer pathogenesis. Nat Commun.

[22] Sun X, Xin S, Zhang Y, Jin L, Liu X, Zhang J (2024). [Corrigendum] Long non-coding RNA CASC11 interacts with YBX1 to promote prostate cancer progression by suppressing the p53 pathway. Int J Oncol.

[23] Xu Q, Qiao H, Xu Y, Zhao Y, He N, Zhao J (2023). HSP90 and noncoding RNAs. DNA Cell Biol.

[24] Gupta S, Silveira DA, Piedade GPS, Ostrowski MP, Mombach JCM, Hashimoto RF (2024). A dynamic Boolean network reveals that the BMI1 and MALAT1 axis is associated with drug resistance by limiting miR-145-5p in non-small cell lung cancer. Noncoding RNA Res.

[25] Malakar P, Shukla S, Mondal M, Kar RK, Siddiqui JA (2024). The nexus of long noncoding RNAs, splicing factors, alternative splicing and their modulations. RNA Biol.

[26] Wu Z, Jiang S, Chen Y (2024). Non-coding RNA and drug resistance in cholangiocarcinoma. Noncoding RNA Res.

[27] Hashemi M, Daneii P, Zandieh MA, Raesi R, Zahmatkesh N, Bayat M (2024). Non-coding RNA-Mediated N6-Methyladenosine (m(6)A) deposition: a pivotal regulator of cancer, impacting key signaling pathways in carcinogenesis and therapy response. Noncoding RNA Res.

[28] Wu J, Zhu S, Lin R, Cai W, Lin H, Wu J (2024). LINC00887 regulates malignant progression and T-cell chemotaxis in clear cell renal cell carcinoma by activating CD70 via recruitment of SPI1. Gene.

[29] Zhu Y, Jin L, Shi R, Li J, Wang Y, Zhang L (2022). The long noncoding RNA glycoLINC assembles a lower glycolytic metabolon to promote glycolysis. Mol Cell.

[30] Feng YC, Zhao XH, Teng L, Thorne RF, Jin L, Zhang XD (2020). The pan-cancer lncRNA MILIP links c-Myc to p53 repression. Mol Cell Oncol.

[31] Yang X, Wen Y, Liu S, Duan L, Liu T, Tong Z (2022). LCDR regulates the integrity of lysosomal membrane by hnRNP K-stabilized LAPTM5 transcript and promotes cell survival. Proc Natl Acad Sci U S A.

[32] Baty F, Facompre M, Kaiser S, Schumacher M, Pless M, Bubendorf L (2010). Gene profiling of clinical routine biopsies and prediction of survival in non-small cell lung cancer. Am J Respir Crit Care Med.

[33] Tabatabaee A, Nafari B, Farhang A, Hariri A, Khosravi A, Zarabi A, et al. Targeting vimentin: a multi faceted approach to combat cancer metastasis and drug resistance. Cancer Metastasis Rev. 2023. 10.1007/s10555-023-10154-7. Online ahead of print. 10.1007/s10555-023-10154-7 38012357

[34] Ridge KM, Eriksson JE, Pekny M, Goldman RD (2022). Roles of vimentin in health and disease. Genes Dev.

[35] Wang B, Li J, Li Y, Liang T, Chu X (2022). MiR-630 suppresses non-small cell lung cancer by targeting vimentin. J Clin Lab Anal.

[36] Chen B, Zhang L, Zhou H, Ye W, Luo C, Yang L (2022). HMOX1 promotes lung adenocarcinoma metastasis by affecting macrophages and mitochondrion complexes. Front Oncol.

[37] Shi K, Yang S, Chen C, Shao B, Guo Y, Wu X (2022). RNA methylation-mediated LINC01559 suppresses colorectal cancer progression by regulating the miR-106b-5p/PTEN axis. Int J Biol Sci.

[38] Li H, Liu J, Lai Y, Huang S, Zheng L, Fan N (2021). LINC01559 promotes colorectal cancer via sponging miR-1343-3p to modulate PARP1/PTEN/AKT pathway. Pathol Res Pract.

[39] Lou C, Zhao J, Gu Y, Li Q, Tang S, Wu Y (2020). LINC01559 accelerates pancreatic cancer cell proliferation and migration through YAP-mediated pathway. J Cell Physiol.

[40] Shen H, Zhu H, Chen Y, Shen Z, Qiu W, Qian C (2021). ZEB1-induced LINC01559 expedites cell proliferation, migration and EMT process in gastric cancer through recruiting IGF2BP2 to stabilize ZEB1 expression. Cell Death Dis.

[41] Yang X, Yang Y, Qian X, Xu X, Lv P (2022). Long non-coding RNA LINC01559 serves as a competing endogenous RNA accelerating triple-negative breast cancer progression. Biomed J.

[42] Dong S, Fu Y, Yang K, Zhang X, Miao R, Long Y (2021). Linc01559 served as a potential oncogene and promoted resistance of hepatocellular carcinoma to oxaliplatin by directly sponging miR-6783-3p. Anticancer Agents Med Chem.

[43] Chen X, Wang J, Xie F, Mou T, Zhong P, Hua H (2020). Long noncoding RNA LINC01559 promotes pancreatic cancer progression by acting as a competing endogenous RNA of miR-1343-3p to upregulate RAF1 expression. Aging (Albany NY).

[44] Lin W, Mo CQ, Kong LJ, Chen L, Wu KL, Wu X (2023). FTO-mediated epigenetic upregulation of LINC01559 confers cell resistance to docetaxel in breast carcinoma by suppressing miR-1343-3p. Kaohsiung J Med Sci.

[45] Wang KC, Chang HY (2011). Molecular mechanisms of long noncoding RNAs. Mol Cell.

[46] Feng YC, Liu XY, Teng L, Ji Q, Wu Y, Li JM (2020). c-Myc inactivation of p53 through the pan-cancer lncRNA MILIP drives cancer pathogenesis. Nat Commun.

[47] Wang L, Bo X, Yi X, Xiao X, Zheng Q, Ma L (2020). Exosome-transferred LINC01559 promotes the progression of gastric cancer via PI3K/AKT signaling pathway. Cell Death Dis.

[48] Hu WM, Li M, Ning JZ, Tang YQ, Song TB, Li LZ (2023). FAM171B stabilizes vimentin and enhances CCL2-mediated TAM infiltration to promote bladder cancer progression. J Exp Clin Cancer Res.

[49] Jiang Y, Feng Y, Huang J, Huang Z, Tan R, Li T (2023). LAD1 promotes malignant progression by diminishing ubiquitin-dependent degradation of vimentin in gastric cancer. J Transl Med.

[50] Zou H, Yang Z, Chan YS, Yeung SA, Alam MK, Si T (2022). Single cell analysis of mechanical properties and EMT-related gene expression profiles in cancer fingers. iScience.

[51] Manfioletti G, Fedele M (2022). Epithelial-Mesenchymal Transition (EMT) 2021. Int J Mol Sci.

[52] Mohebi M, Ghafouri-Fard S, Modarressi MH, Dashti S, Zekri A, Kholghi-Oskooei V (2020). Expression analysis of vimentin and the related lncRNA network in breast cancer. Exp Mol Pathol.

[53] Zeng S, Xie X, Xiao YF, Tang B, Hu CJ, Wang SM (2018). Long noncoding RNA LINC00675 enhances phosphorylation of vimentin on Ser83 to suppress gastric cancer progression. Cancer Lett.

