# Supplementary material for: LINC01559 promotes lung adenocarcinoma metastasis by disrupting the ubiquitination of vimentin
Source: Biomark Res. 2024 Feb 5;12:19. doi: 10.1186/s40364-024-00571-3 (PMC10840222; doi:10.1186/s40364-024-00571-3)
Supplement: Supplementary file 1 — Additional file 1: Supplementary Figure 1. Overexpression of LINC01559 promotes LUAD metastasis. [file 40364_2024_571_MOESM1_ESM.pdf]

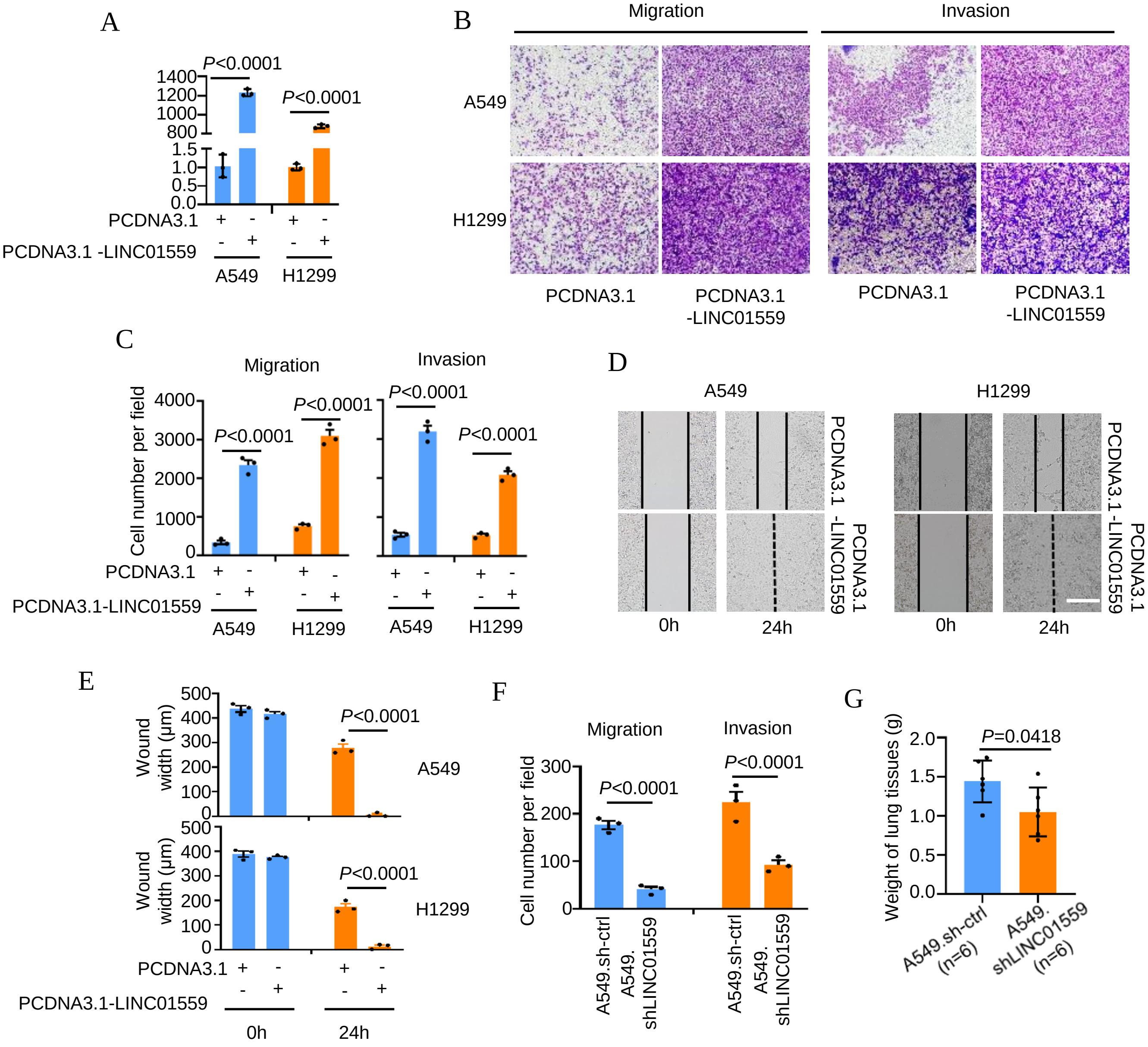

Supplementary Figure 1. Overexpression of LINC01559 promotes LUAD metastasis. (A-E) Overexpression of LINC01559 (A) promoted migration and invasion (**B, C**) and wound healing (**D, E**) of A549 and H1299 cells. The numbers of migrated and invasive cells were quantitated using the ImageJ software. Scale bars, 200 µm (wound healing assay) and 200 µm (cell migration and invasion assay). Data are mean ± s.d.; n = 3 independent experiments, one-way ANOVA followed by Tukey's multiple comparisons test. (**F**) ShRNA knockdown of LINC01559 inhibited migration and invasion of A549 cells. Data are mean ± s.d.; n = 3 independent experiments, one-way ANOVA followed by Tukey's multiple comparisons test. (**G**) The lungs of NOD/SCID mice that received tail vein injection of A549.shLINC01559 cells were lighter than those that received tail vein injection injection of A549.sh-ctrl cells. n=6; Two-tailed Student's t-test.
