# Supplementary material for: LINC01559 promotes lung adenocarcinoma metastasis by disrupting the ubiquitination of vimentin
Source: Biomark Res. 2024 Feb 5;12:19. doi: 10.1186/s40364-024-00571-3 (PMC10840222; doi:10.1186/s40364-024-00571-3)
Supplement: Supplementary file 2 — Additional file 2: Supplementary Figure 2. Overexpression of vimentin promotes LUAD metastasis. [file 40364_2024_571_MOESM2_ESM.pdf]

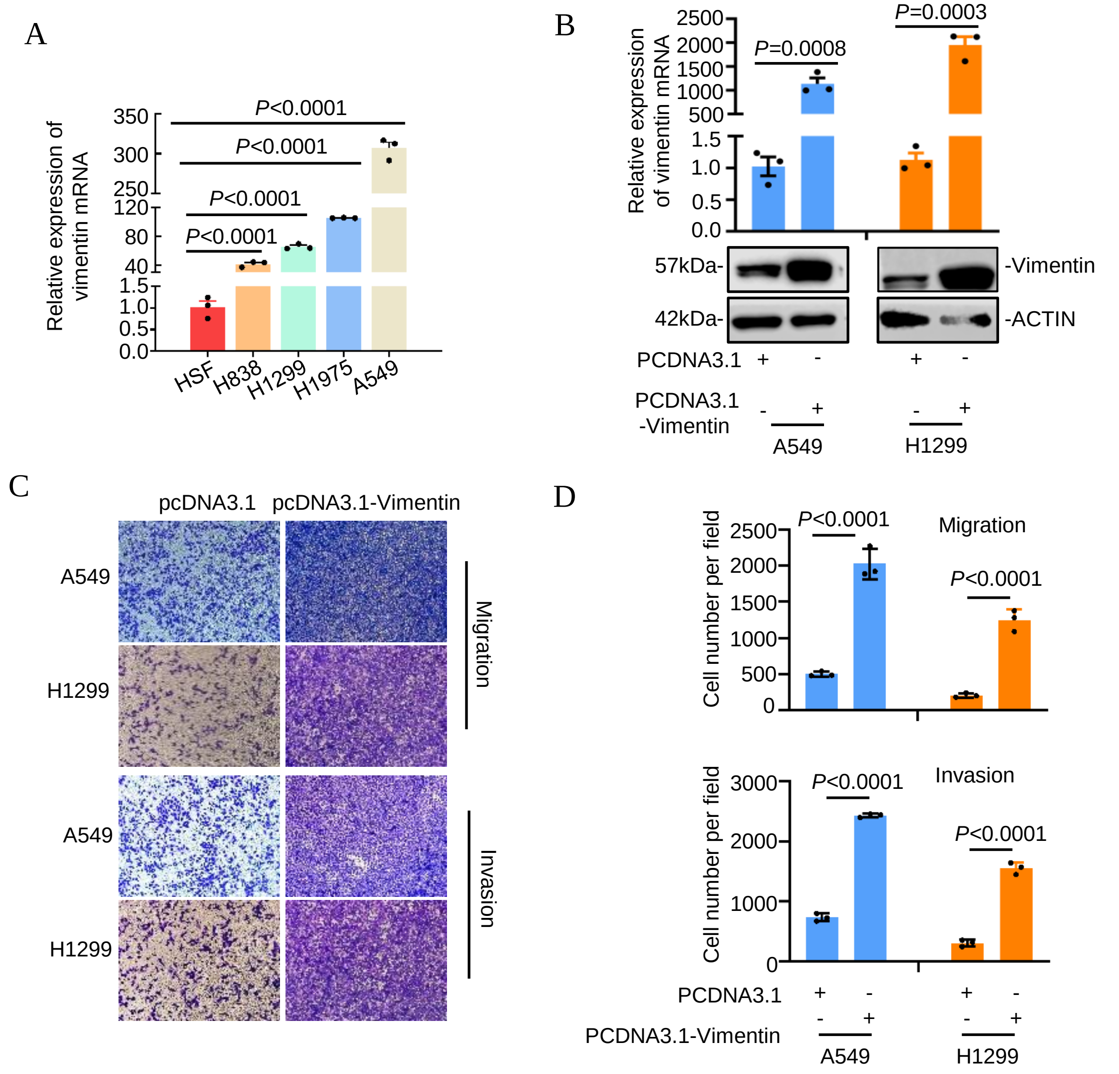

Supplementary Figure 2. Overexpression of vimentin promotes LUAD metastasis. (A) qPCR analysis of vimentin expression in the indicated LUAD cell lines and the human fibroblast cell line HSF. Data are mean ± s.d.; n = 3 independent experiments, one-way ANOVA followed by Tukey's multiple comparisons test. (**B-D**) Overexpression of vimentin (**B**) promoted migration and invasion (**C, D**) of A549 and H1299 cells. Scale bars 200μm. Data are mean ± s.d.; n = 3 independent experiments, one-way ANOVA followed by Tukey's multiple comparisons test.
