# Supplementary material for: LINC01559 promotes lung adenocarcinoma metastasis by disrupting the ubiquitination of vimentin
Source: Biomark Res. 2024 Feb 5;12:19. doi: 10.1186/s40364-024-00571-3 (PMC10840222; doi:10.1186/s40364-024-00571-3)
Supplement: Supplementary file 3 — Additional file 3: Supplementary Table 1. Primer sequences of quantitative real-time PCR. [file 40364_2024_571_MOESM3_ESM.docx]

**Supplementary Table 1**: Primer sequences of quantitative real-time PCR.

| Gene | Sequences 5'-3' |
| --- | --- |
| LINC01559 | (Forward) TCCCTCAGCCAAGTCCTTCCTTAC |
|  | (Reverse) GTCCAGTTCATGCTCTGACAGTCC |
| Vimentin | (Forward)AGGCAAAGCAGGAGTCCACTGA |
|  | (Reverse) ATCTGGCGTTCCAGGGACTCAT |
| **β**-actin | (Forward) CTGGGACGACATGGAGAAAA |
|  | (Reverse) AAGGAAGGCTGGAAGAGTGC |
| U1 | (Forward) GGCGAGGCTTATCCATTG |
|  | (Reverse) CCCACTACCACAAATTATGC |
