# Supplementary material for: LINC01559 promotes lung adenocarcinoma metastasis by disrupting the ubiquitination of vimentin
Source: Biomark Res. 2024 Feb 5;12:19. doi: 10.1186/s40364-024-00571-3 (PMC10840222; doi:10.1186/s40364-024-00571-3)
Supplement: Supplementary file 4 — Additional file 4: Supplementary Table 2. Summary of proteins that interact with LINC01559 in A549 cells detected using mass spectrometry. [file 40364_2024_571_MOESM4_ESM.docx]

**Supplementary Table 2.** Summary of proteins that interact with LINC01559 in A549 cells

detected using mass spectrometry

| No. | Entry name | Coverage (%) | MW (kDa) | Score |
| --- | --- | --- | --- | --- |
| 1 | VIM | 61 | 53.6 | 146.56 |
| 2 | KRT1 | 51 | 66.0 | 142.91 |
| 3 | YBX3 | 61 | 40.1 | 110.39 |
| 4 | NCL | 34 | 76.6 | 109.78 |
| 5 | KRT2 | 28 | 65.4 | 84.48 |
| 6 | KRT18 | 67 | 48.0 | 83.85 |
| 7 | KRT5 | 14 | 62.3 | 82.35 |
| 8 | KRT10 | 42 | 58.8 | 82.07 |
| 9 | RPA1 | 41 | 68.1 | 81.96 |
| 10  11  12  13  14  15  16  17  18  19  20  21 | TUBB  TUBB4B  TUBB2A  PCCB  TUBA1A  KRT9  ATP5B  ATP5A1  TUBA4A  TUBB6  DAZAP1  HNRNPD | 54  53  41  44  37  33  39  34  31  16  24  35 | 49.6  49.8  49.9  58.2  50.1  62.0  56.5  59.7  49.9  49.8  43.4  38.4 | 80.97  75.32  64.92  53.49  52.31  46.92  45.89  39.95  39.71  35.43  30.41  30.11 |
