# Supplementary material for: LINC01559 promotes lung adenocarcinoma metastasis by disrupting the ubiquitination of vimentin
Source: Biomark Res. 2024 Feb 5;12:19. doi: 10.1186/s40364-024-00571-3 (PMC10840222; doi:10.1186/s40364-024-00571-3)

Fig. 3C gels of A549 and H1299

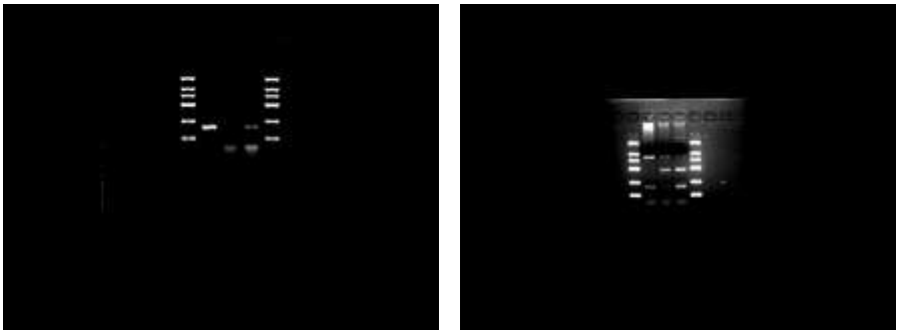

Fig. 3C blots of A549 and H1299 (The right lane is the result of other experiments)

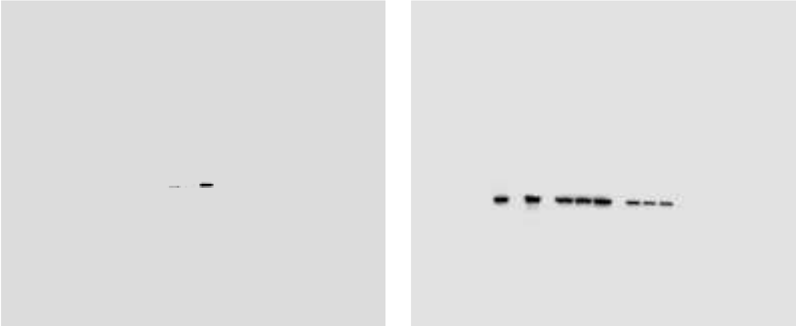

Fig. 3D gels of A549 and H1299

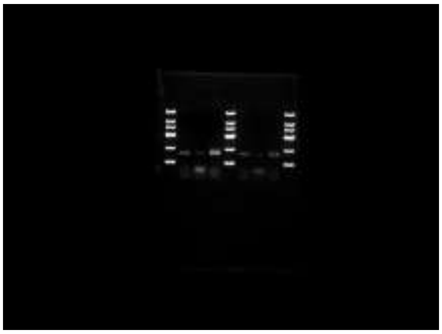

Fig. 3D blots of A549 and H1299

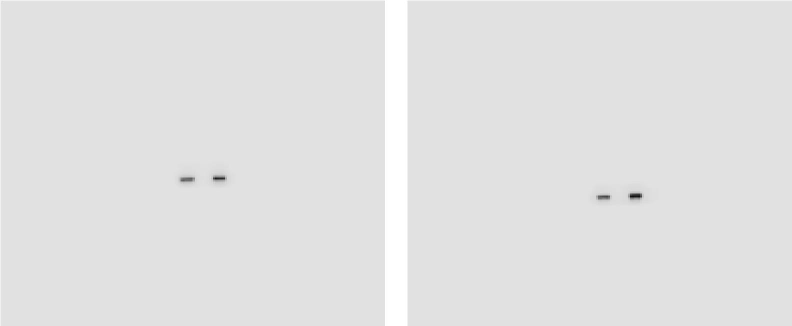

Fig. 4A blots of A549 and H1299 (The fourth lane on the right side of the A549 image is siVIM-3. We have reserved siVIM-1 and siVIM-2 as needed)

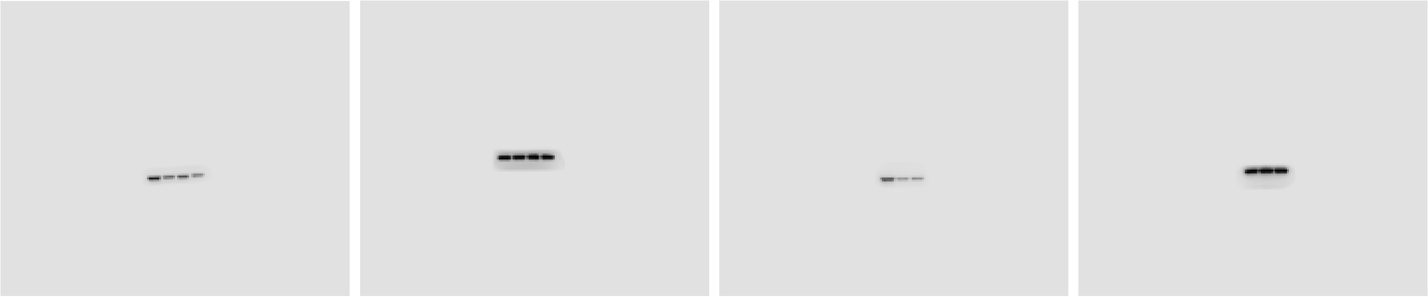

Fig. 5B blots of A549 and H1299

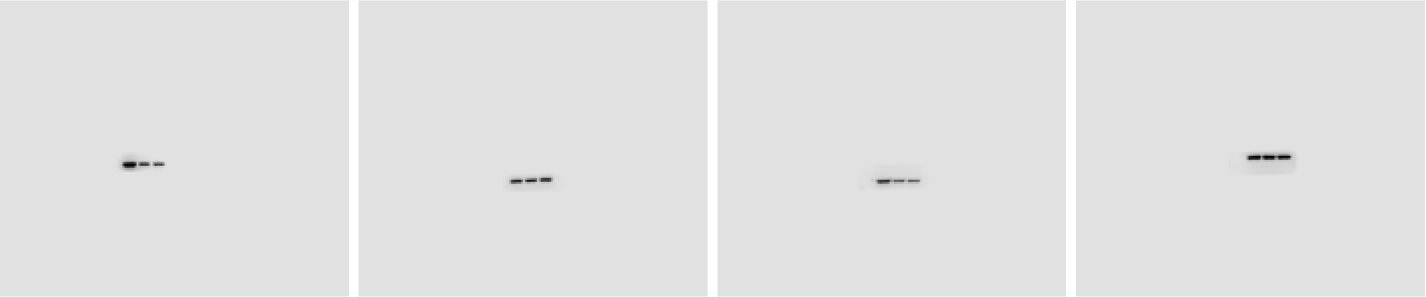

Fig. 5D blots of A549 and H1299

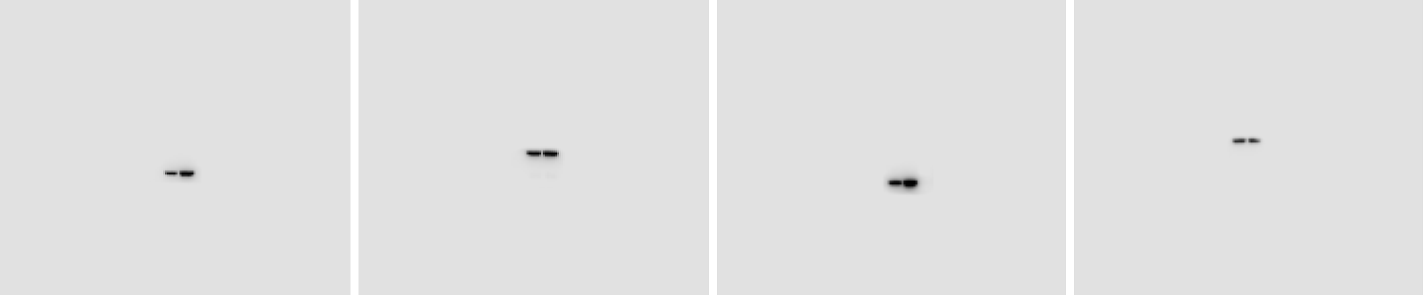

Fig. 5G blots

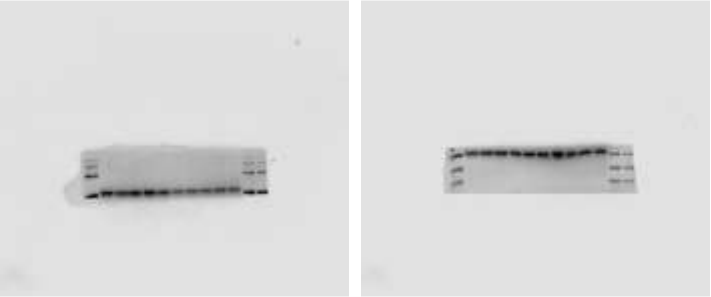

Fig. 6A blots of A549 and H1299

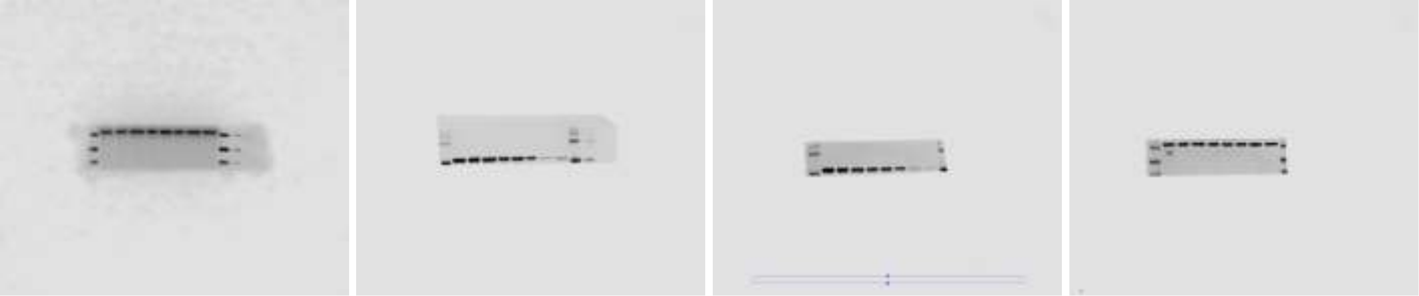

Fig. 6B blots of A549 and H1299

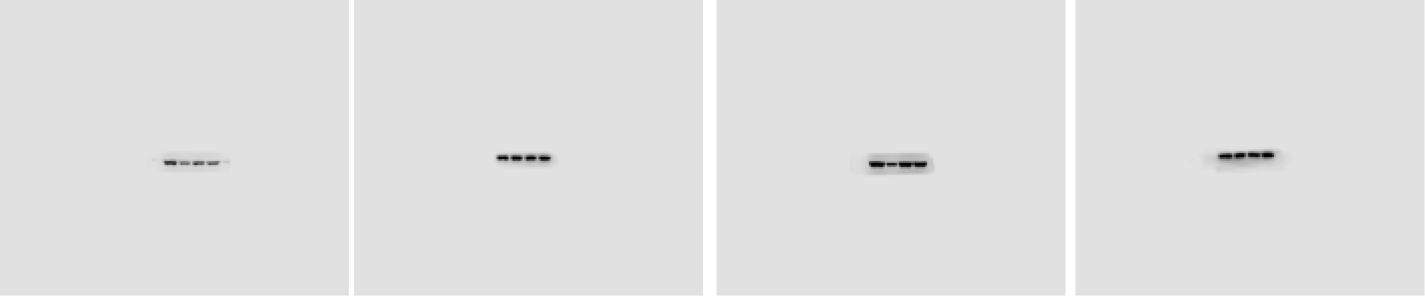

Fig. 6C blots of A549 and H1299

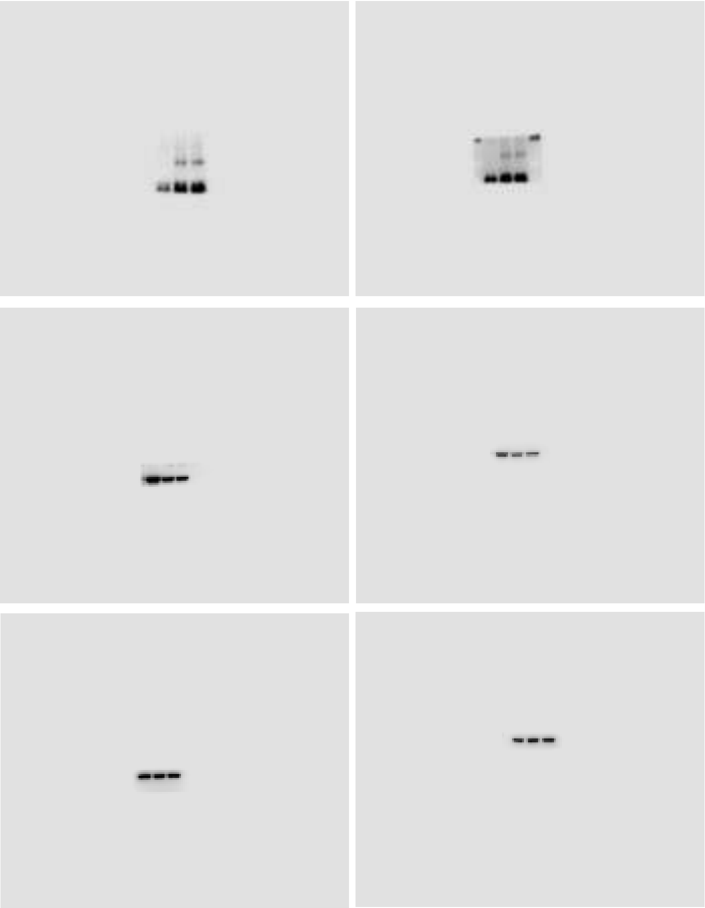

Fig. 6D blots of A549 and H1299

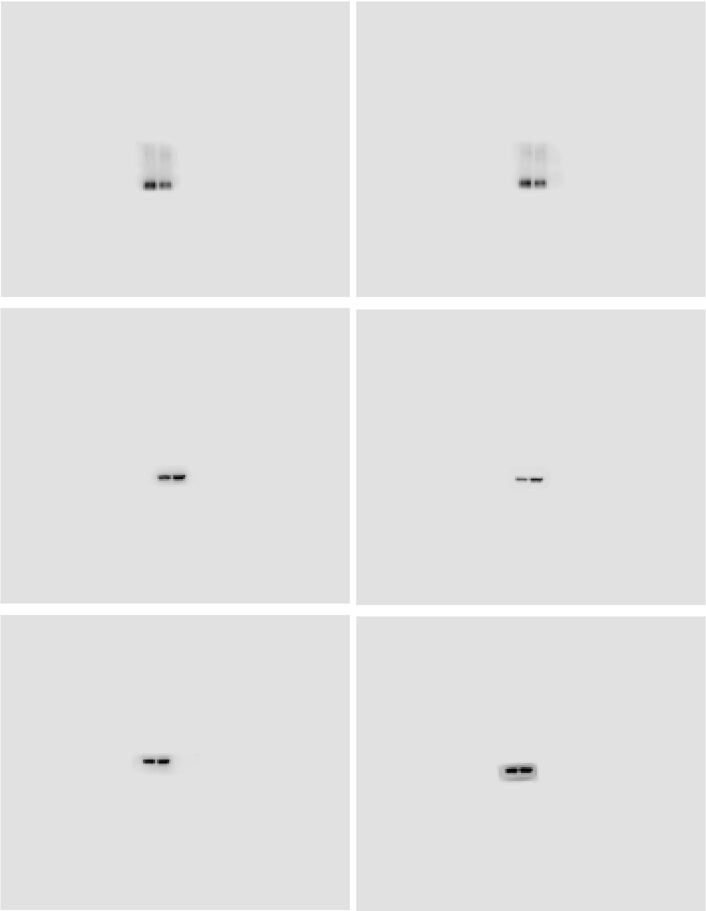

Fig. 6E blots

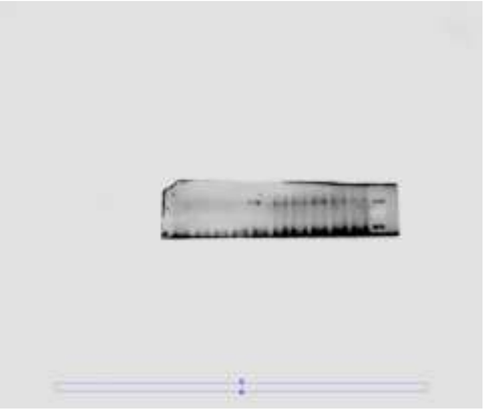

Supplementary Figure. 2B blots

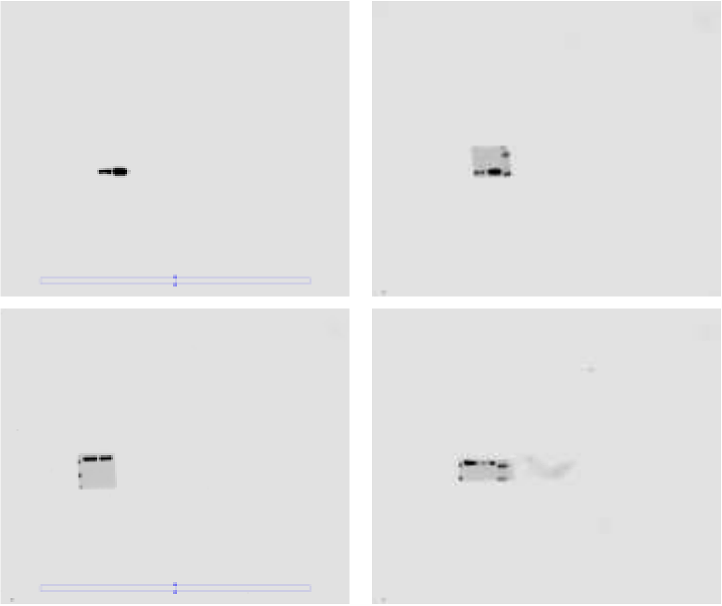

Supplement: Supplementary file 5 — Additional file 5. [file 40364_2024_571_MOESM5_ESM.pdf]
